# Multiresolution analysis for COVID-19 diagnosis from chest CT images: wavelet vs. contourlet transforms

**DOI:** 10.1007/s11042-023-15485-9

**Published:** 2023-05-12

**Authors:** Lamiaa Abdel-Hamid

**Affiliations:** Department of Electronics & Communication, Faculty of Engineering, Misr International University (MIU), Cairo, Egypt

**Keywords:** COVID-19, Chest CT images, Classification, Contourlet transform, Wavelet transform

## Abstract

Chest computer tomography (CT) provides a readily available and efficient tool for COVID-19 diagnosis. Wavelet and contourlet transforms have the advantages of being localized in both space and time. In addition, multiresolution analysis allows for the separation of relevant image information in the different subbands. In the present study, transform-based features were investigated for COVID-19 classification using chest CT images. Several textural and statistical features were computed from the approximation and detail subbands in order to fully capture disease symptoms in the chest CT images. Initially, multiresolution analysis was performed considering three different wavelet and contourlet levels to determine the transform and decomposition level most suitable for feature extraction. Analysis showed that contourlet features computed from the first decomposition level (L1) led to the most reliable COVID-19 classification results. The complete feature vector was computed in less than 25 ms for a single image having of resolution 256 × 256 pixels. Next, particle swarm optimization (PSO) was implemented to find the best set of L1-Contourlet features for enhanced performance. Accuracy, sensitivity, specificity, precision, and F-score of a 100% were achieved by the reduced feature set using the support vector machine (SVM) classifier. The presented contourlet-based COVID-19 detection method was also shown to outperform several state-of-the-art deep learning approaches from literature. The present study demonstrates the reliability of transform-based features for COVID-19 detection with the advantage of reduced computational complexity. Transform-based features are thus suitable for integration within real-time automatic screening systems used for the initial screening of COVID-19.

## Introduction

Coronavirus (COVID-19) is a highly contagious, severe respiratory disease that spreads more rapidly than the traditional flu. On March 11, 2020, the World Health Organization (WHO) declared COVID-19 as a global pandemic [21]. Since its outbreak, COVID-19 has affected millions of people worldwide. By September 2022, it has sadly caused the death of over 6.5 million persons around the globe [19].

COVID-19 symptoms include fever, dry cough, shortness of breath, chest pain, fatigue, loss of taste, loss of smell, headaches, sore throat, runny nose, diarrhea, abdominal pain, and nausea [13]. Most COVID-19 patients tend to have mild or moderate flu-like symptoms, whereas few suffer from severe respiratory problems. Some patients, however, are healthy or lucky enough not to notice any symptoms. In mild and moderate cases, lung lesions are typically absorbed after treatment. Yet in advanced cases, lung damage may be severe and irreversible. In addition, moderate disease symptoms left untreated were sometimes found to rapidly progress causing severe complications that lead to hospitalization, lengthy recovery periods, and problems with lung and heart functions [20]. Early detection and proper treatment of COVID-19 is thus essential to speed up recovery and lessen the odds of developing severe complications, by that reducing the demand on the healthcare system and saving lives.

Shortly after the start of the outbreak, polymerase chain reaction (PCR) tests became the gold standard for COVID-19 diagnosis. PCR works by detecting the genetic material of the virus on a swab taken from a person’s nose and/or throat. PCR tests require highly trained professionals and sophisticated lab equipment which can be a challenge in suburban or rural regions. In developing countries, PCR tests are also considerably expensive and not abundantly available. In addition, PCR tests suffer from low sensitivity, meaning that there is high probability that a COVID-19 positive case would be missed by the test [64].

Chest computer tomography (CT) has thus rapidly emerged as a valuable tool for the diagnosis and follow-up of COVID-19 patients, especially those exhibiting acute respiratory symptoms. CT scan equipment are already readily available and commonly utilized in many medical institutions worldwide. In addition, CT scans were shown to have a higher sensitivity than PCR tests [40]. However, accurate diagnosis using chest CT scans requires their thorough examination by an experienced personal, a task that would cause a huge overload on the limited available medical experts. Computer-aided-diagnosis (CAD) has the potential to reduce the load on the healthcare system by providing an automatic, fast, and reliable method for COVID-19 screening from the chest CT images.

**Fig. 1:**
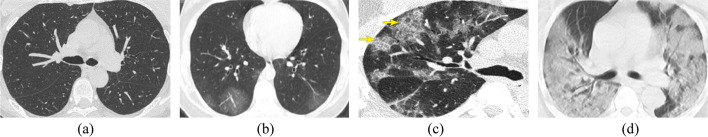
Examples of chest CT images showing **a** no COVID-19 symptoms [52], **b** ground glass opacity (GGO) [26], **c** crazy paving (pointed to by the yellow arrows) [51], and **d** consolidations [26]

CT image abnormalities in COVID-19 patients vary according to the disease stage and severity. Ground-glass opacites (GGO) are considered the earliest and most common radiological abnormality found in up to 98% of the chest CT scans of COVID-19 patients [9]. GGO are usually described as peripheral and bilateral hazy areas with increased lung opacity through which vessels and bronchial structures can still be seen. At later stages, these opacities become patchy with an irregular paving stones appearance (crazy paving). In addition, consolidations may start to appear which are characterized by their white-dense appearance masking vessels and bronchial structures. Other chest CT manifestations of COVID-19 include reticular pattern, air bronchogram, bronchus deformation, fibrosis, vascular dilation, pulmonary nodules, and halo signs. Figure 1 shows examples of chest CT scans of normal and COVID-19 patients at different disease stages.

Wavelet and contourlet transforms have the advantage of being localized in both space and time, which makes them well suited for medical images analysis. Multiresolution decomposition separates an image’s edge and luminance information in its different subbands, as well as brings out different image related information in the different decomposition levels. In addition, the downsampling step implemented within both transforms allows features to be rapidly computed from the downscaled subbands. The aim of the present study is to investigate the reliability of the wavelet and contourlet transforms for COVID-19 detection from chest CT images. Several transform-based features were thus computed from the approximation and detail subbands in order to fully capture the COVID-19 symptoms within the chest CT images. Various analyses were then performed to determine the transform and decomposition level most suitable for COVID-19 classification. Next, feature selection was implemented to find the best set of features for optimal classification performance. The introduced transform-based COVID-19 detection method is shown to outperform several state-of-the-art deep learning approaches from literature, with the added advantage of reduced computational complexity. The merits of the proposed method can be attributed to the efficiency of the implemented transform-based features in capturing COVID-19 manifestations in the chest images, in addition to them being rapidly computed from the downsampled transform subbands.

The rest of the paper is organized as follows: Section 2 describes several related works considering both traditional and deep learning approaches. Section 3 gives a brief summary of multiresolution analysis techniques, specifically the wavelet and contourlet transforms considered in this work. Section 4 introduces the two public chest CT images datasets utilized in the present study, as well as the transform-based features computed for COVID-19 detection. Section 5 summarizes the performance metrics used to evaluate the performance of the introduced algorithm. In Section 6, the results of all experiments are presented and discussed in addition to being compared to various state-of-the-art methods from literature. Section 7 presents a discussion of the merits and limitations of the introduced transform-based COVID-19 detection method. Finally, Section 8 draws the conclusions of this study, then gives directions for future work.

## Literature review

Artificial intelligence (AI) approaches for the detection of COVID-19 from chest CT images can be divided into traditional and deep learning approaches. Traditional COVID-19 detection methods consider handcrafted features computed either from the spatial or transform domain of the chest CT images. Examples of features used include statistical (e.g., mean, variance, skewness, kurtosis, energy, entropy, etc.) and textural features (e.g., gray level co-occurrence matrices (GLCM), local binary patterns (LBP), etc.). Chen [14] considered GLCM features for COVID-19 detection from chest CT images using a support vector machine (SVM) classifier. For a small dataset of 296 images, an accuracy of 75.69% was reported. Tan et al. [59] initially calculated 1,688 textural, statistical, and shape features. Next, they implemented feature selection to remove irrelevant features. They tested their algorithm on a dataset consisting of 319 chest CT images achieving accuracies of approximately 98 ± 1% in their different experiments. Both of these approaches have the limitation of being tested on very small datasets (< 350 images), in addition to achieving limited performance. Although promising, handcrafted-based COVID-19 detection methods, so far, leave a huge room for improvement.

Unlike traditional methods that rely on an expert for the extraction of relevant handcrafted features, deep learning methods incrementally learn high-level distinctive features from the data without the need for any human intervention. Convolutional neural networks (CNNs) are typically used deep learning networks for image processing and classification. Deep networks used for image classification can be categorized into standard pre-trained networks and customized networks.

Pre-trained networks refer to standard CNNs that have been previously introduced and trained on a large generic dataset. ImageNet [22] is the most commonly used dataset for pretraining standard CNNs as it consists of 1.2 million images within 1,000 categories. These pre-trained networks are then fine-tuned using the target dataset for the final classification. This process is commonly referred to as transfer learning (TL). TL has the advantage leveraging knowledge from one domain where abundant data is available to another domain having limited data hence boosting the latter’s performance. Due to the complexity, cost, and ethical issues associated with data collection, medical datasets are typically much smaller than those used for computer vision tasks (e.g., hundreds/thousands vs. millions) [43]. TL is thus widely applied in the medical domain where limited data is available.

COVID-19 detection approaches relying on TL techniques having been widely implemented in literature considering various networks such as AlexNet, VGG, ResNet, MobileNet, Inception, and DenseNet [6, 45]. Ahuja et al. [7] compared three pre-trained variants of ResNet to SqueezeNet for COVID-19 detection. ResNet18 achieved the best results with an area under the receiver operating characteristics curve (AUC) of 0.996 and an accuracy of 99.4%. Jaiswal et al. [38] compared TL approaches using VGG16, Inception-ResNetV2, ResNet152V2, and DenseNet. They concluded that, for the considered networks, DenseNet gave the highest results (AUC = 0.97 and accuracy = 96.25%). Gaur et al. [31] also considered TL, but the novelty of their work was that they utilized the empirical wavelet transform for decomposing the chest CT images. Wavelet subbands from levels 1 to 5 were applied as inputs to several DenseNet networks. Best results achieved were for the DenseNe121 (AUC = 0.96 and accuracy = 85.5%). Several other works have also compared different deep architectures [30, 44, 57] where, interestingly, the best performing pre-trained network was found to vary across the different research. Another limitation of TL approaches is that they are somewhat generic. TL methods consider pre-trained deep networks that were originally designed for computer vision tasks, not for medical image analysis. By that, these networks may not efficiently take into account the anatomical structures within the analyzed medical images, which could in turn affect its overall performance.

Although there has been an abundant body of research relying on TL for COVID-19 detection from chest CT images, limited works implemented customized networks for the task. Among these works, Pathak et al. [49] used a deep bidirectional long short-term memory network with mixture density network (DBM). In their work, they tune the DBM’s hyperparameters using a Memetic Adaptive Differential Evolution (MADE) algorithm resulting in an accuracy of 98.37% for a large public dataset having 2,482 chest CT images. Another work by Islam et al. [36] introduced a novel deep CNN for extracting prominent features from the chest CT images. They used an ensemble learner based on five different classifiers and soft voting to detect COVID-19 resulting in accuracy of 99.73%. Both these works showed that their deep learning methods outperformed various TL-based approaches from literature. These findings are in agreement with previous research that showed that in medical applications, a well-developed deep network can significantly outperform generic TL based methods [3, 43].

An interesting study by Dina et al. [53] combines both deep and handcrafted features for COVID-19 disease diagnosis. Pre-trained AlexNet, GoogleNet, ShuffleNet, and ResNet18 were all employed to extract the deep features. Statistical and textural features along with different wavelet subbands were considered as the handcrafted features. The authors then compared using (1) deep features only (6,176 feature), (2) handcrafted features only (772 feature), and (3) FUSI-CAD were both the deep and handcrafted features were combined. FUSI-CAD was shown to give the highest results considering a public dataset of 2,482 chest CT images. Interestingly, the difference in performance between the three compared scenarios was minimal. For example, FUSI-CAD’s AUC and accuracy were higher than for the case of using only the handcrafted features by merely 1% and 0.5%, respectively. Their work thus showed that handcrafted features have great potential for the development of reliable COVID-19 detection algorithms with the added advantage of reduced computation complexity and less data requirements [60].

COVID-19 automatic detection methods in literature mainly considered the spatial chest images for feature computation. Multiresolution decomposition is another approach for image representation that has been successfully implemented for various disease classifications from CT images [41, 50]. Multiresolution analysis has the ability to separate an image’s luminance (low frequency) and edge (high frequency) information in its different subbands, such that each can be analyzed and processed separately. Although Dina et al. [53] have previously considered wavelet features in their algorithm, the wavelet subband coefficients were directly used as features as opposed to extracting relevant handcrafted measures from them. In addition, wavelet coefficients were used alongside other spatial and deep features by that not fully exploring the potential of transform-based features for the implementation of reliable automatic COVID-19 diagnosis algorithms.

In the present study, a transform-based COVID-19 detection algorithm from chest CT images is proposed that relies solely on handcrafted features. This work was inspired by two main research papers: The first is the work of Dina et al. [53] which showed that for COVID-19 detection from chest CT images, handcrafted features gave results that were almost as good as those attained by deep features. The second was a previous work by the author in which transform-based features were found to significantly outperform several other spatial features for disease diagnosis [2]. The aim of the present study is thus to investigate the usefulness of handcrafted transform-based features for the development of a computationally efficient, fast, and reliable COVID-19 detection algorithm from chest CT images. The main contributions of this paper can be summarized as follows:

1. Different handcrafted transform-based features were calculated from the approximation and detail subbands in order to fully capture COVID-19 symptoms in the CT images.
2. A comprehensive analysis was made to determine the transform (wavelet vs. contourlet) and decomposition level better suited for COVID-19 classification.
3. Following the previous analysis, feature selection was implemented to find the best set of transform-based features for optimal performance.
4. A computationally efficient, fast, and reliable transform-based COVID-19 diagnosis algorithm was finally presented and shown to outperform several state-of-the art deep learning methods with the advantage of being computationally inexpensive.

## Multiresolution transforms

Multiresolution decomposition has the merit of being similar to the human visual system (HVS) which indicates that the eye decomposes images into low and high frequency subbands then analyzes each differently [29]. The decomposition of images into several scales and directions assists in their deep analysis, leading in turn to relevant feature extraction from the different subbands. In addition, multiresolution techniques typically involve image dimensionality reduction allowing for reduced computation complexity and faster feature computation [4]. Among the most widely used transforms in image processing applications are the wavelet and contourlet transforms, both which have the advantage of being localized in both space and time.

### Wavelet transform

**Fig. 2:**
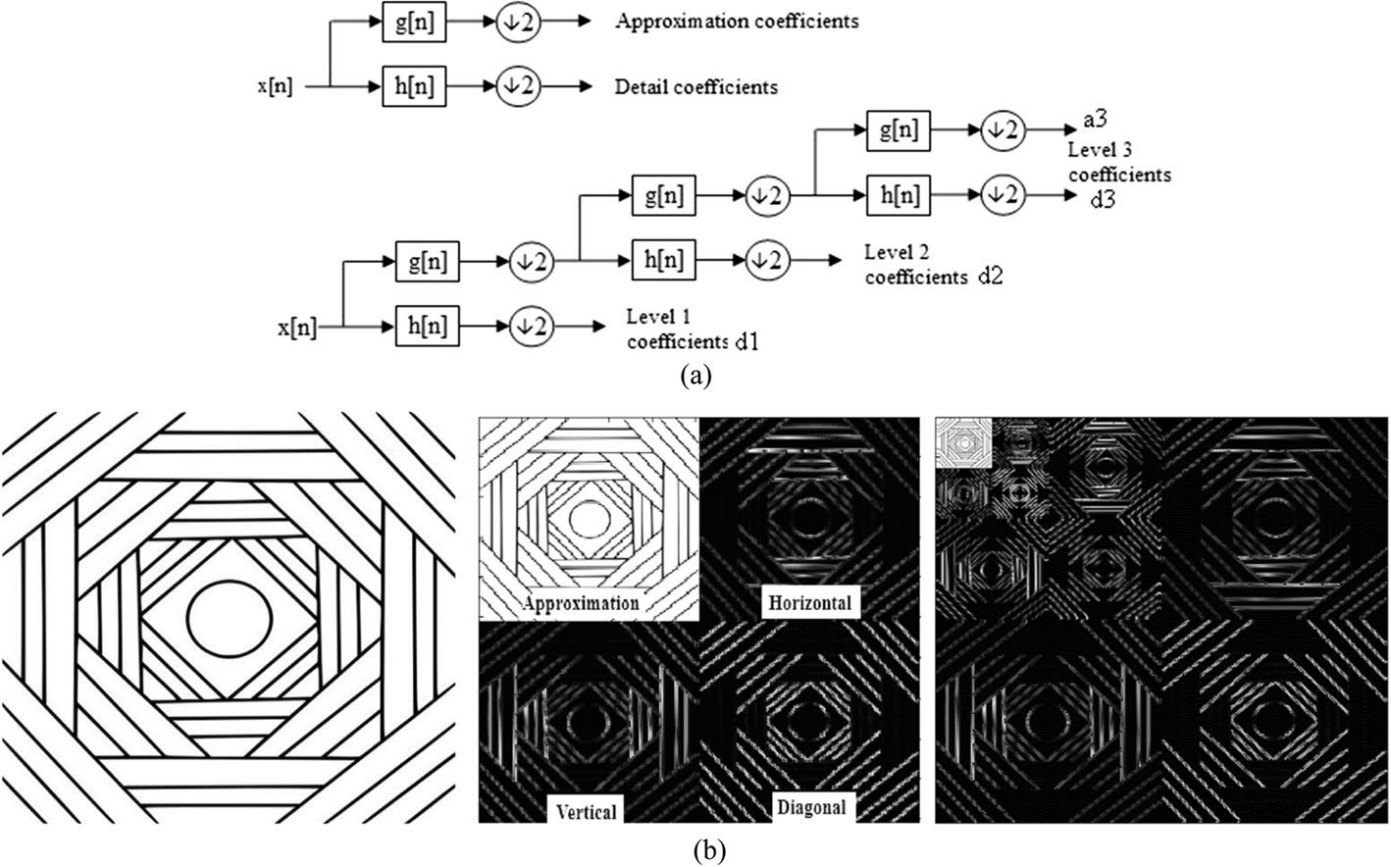
**a** Wavelet transform multiresolution decomposition scheme [33], **b** From left to right: Original image—single wavelet decomposition indicating the approximation and detail (horizontal – vertical – diagonal) subbands—three level multiresolution wavelet decomposition [1]

Wavelet transform (WT) [5] applies a series of low (L) and high (H) pass filters along the rows and columns of an image to separate its illumination and edge information in the approximation (LL) and details subbands, respectively. The high frequency information is distributed among three different detail subbands representing an image’s horizontal (LH), vertical (HL), and diagonal (HH) edge information. Multiresolution analysis can be implemented by reapplying the WT to an image’s approximation subband by that bringing out its finer details in higher decomposition levels. More details concerning the mathematical formulations of the WT can be found in Ref. [27]. Figure 2a shows the block diagram of a single-level wavelet decomposition, whereas Fig. 2b demonstrates different wavelet subbands for single and multilevel wavelet decompositions of a sample image.

### Contourlet transform

**Fig. 3:**
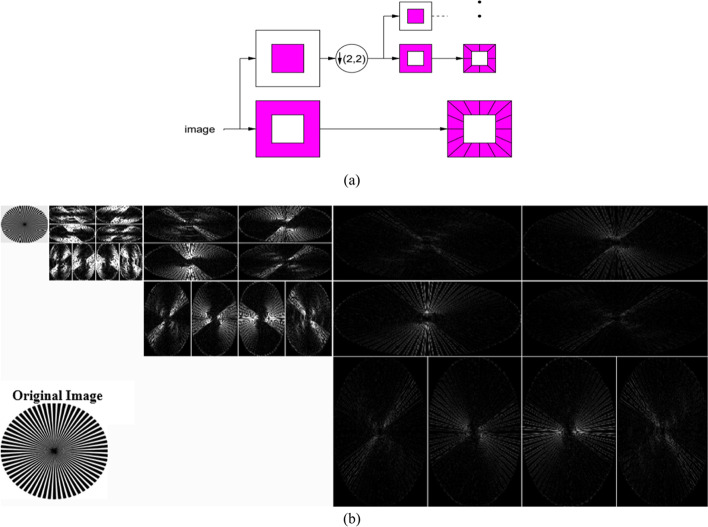
**a** Contourlet transform multiresolution decomposition scheme [24], **b** Original image along with its three level contourlet decomposition, where the detail subbands in all levels are decomposed into eight different directions

A drawback of the wavelet transform is its limited capability to efficiently capture directional information within the images, specifically for curved structures. Contourlet transform [24] uses a double filter bank structure to get smooth image contours. Initially, the image is decomposed into low and high frequency subbands. Next, directional filter banks (DFBs) are applied to the high frequency subbands in order to further bring out the image’s finer details. Furthermore, multiresolution analysis can be implemented by reapplying the contourlet transform to the approximation subband by that bringing out its finer details in the higher decomposition levels. More details on the mathematical representation of the contourlet transform can be found in Ref. [24]. Figure 3a illustrates the block diagram of a Contourlet filter bank, whereas Fig. 3b shows the different contourlet subbands for the 3-level decomposition of a sample image.

Contourlet transform has several advantages over WT such as directionality and anisotropy. The DFBs oriented at several directions implemented within the contourlet transform allows for an image’s decomposition in more than just the three directions offered by the WT. In addition, the elongated shapes and different aspect ratios of the basic elements within the contourlet transform facilitate the capturing of the smooth contours within images. Such merits generally make the contourlet transform more suitable for curve representation than the WT [24].

## Materials & methods

### Chest CT datasets

Two chest CT datasets were used for the development and testing of the proposed COVID-19 detection algorithm (Table 1):

1. **CoDS1 (SARS-CoV-2)** [57]: includes 1,230 positive and 1,252 negative COVID-19 chest CT scans. Images were collected from several hospitals across Sao Paulo, Brazil.
2. **CoDS2 (subset of iCTCF** [48])**:** consists of 3,777 positive and 3,910 negative COVID-19 chest CT scans. Images were collected from two hospitals: Union Hospital (HUST-UH) and Liyuan hospital (HUST-LH) in China.

**Table 1:** Summary of the two considered COVID-19 chest CT datasets

Dataset	COVID	Non-COVID	Total
CoSD1	1,230	1,252	2,482
CoDS2	3,777	3,910	7,687

**Fig. 4:**
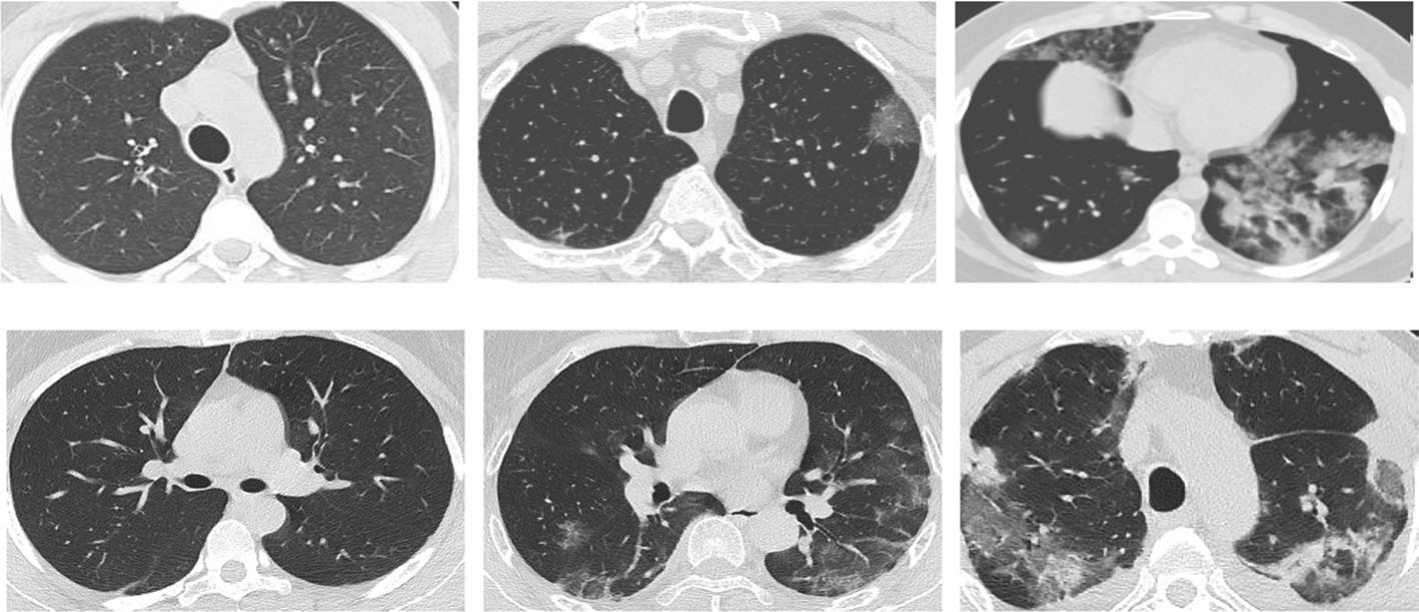
Chest CT sample images of COVID-19 negative  (column 1) and COVID-19 positive (columns 2&3) patients from the CoDS1 (row1) and the cropped CoDS2 (row 2) datasets

Figure 4 shows sample images from the CoDS1 and the CoDS2 chest CT datasets. The first dataset (CoDS1) was used for the preliminary analysis in which the different transforms and decomposition levels were compared. The second larger dataset (CoDS2) was considered for the final classification experiments.

### Preprocessing

**Fig. 5:**
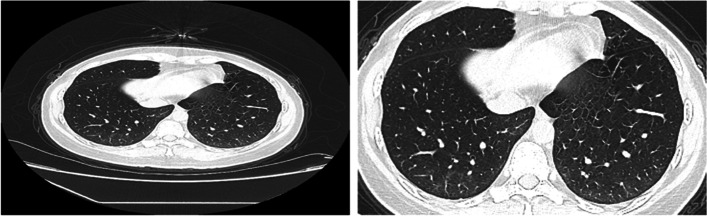
Sample chest CT image from CoDS2 before (left) and after (right) cropping

Chest CT images are typically surrounded by a large black area that includes no relevant diagnostic information. The CoDS1 chest CT images were already cropped by its providers. As for the CoDS2 dataset images, the black region surrounding the chest was cropped using Algorithm 1. Figure 5 shows a sample image from CoDS2 before and after cropping. For both datasets, the cropped chest CT images were resized to 256 × 256 pixels to make them suitable for the contourlet decomposition as well as to assure consistent information extraction from the different images [5]. Finally, contrast stretching was applied to all images in order to enhance their contrast prior to feature extraction. Contrast stretching works by stretching an image’s intensity values to the full range of pixel values [54]. Specifically, the chest CT image intensity values between 0.1 and 0.9 were stretched to the range from 0 to 1 by that accenting the image details for enhanced classification performance.

**Figure:**
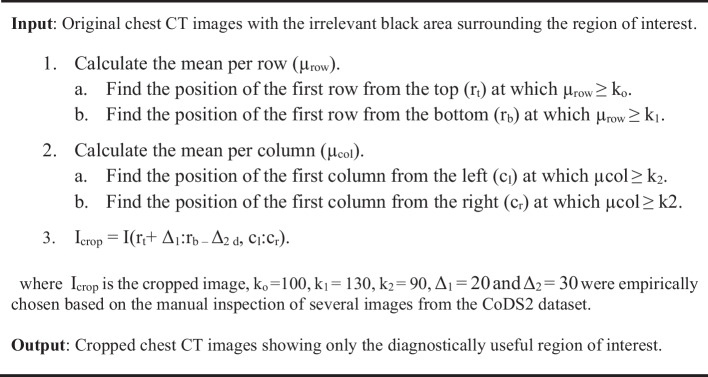
Algorithm 1: Chest CT Image Cropping

### Feature extraction

**Fig. 6:**
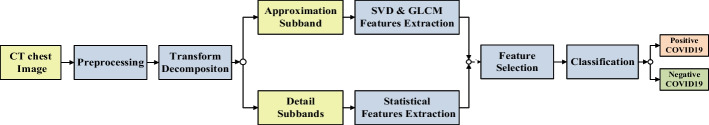
Flowchart of the proposed transform-based COVID-19 detection algorithm from chest CT images

COVID-19 significantly alters the overall appearance and texture of a patient’s chest CT images. Multiresolution decomposition has the advantage of separating an image’s luminance and edge information in its approximation and detail subbands, respectively. In this work, a transform-based COVID-19 detection algorithm is presented in which different handcrafted features are computed from the approximation and detail subbands in order to capture these changes within the chest CT images. Figure 6 shows the flowchart of the introduced transform-based COVID-19 detection algorithm.

#### Singular Value Decomposition (SVD) features

SVD is a robust and reliable orthogonal matrix factorization method. It is considered a linear algebra technique that represents any matrix (A) of size *m* × *n* as the product of three matrices as follows:

1 $$A=U\;S\;V^T$$

where U_*m*×*m*_ and V_*n*×*n*_ are unitary matrices, and S_*m*×*n*_ is a diagonal matrix containing the square roots of the non-zero Eigen values of matrices U and V. The diagonal values in the S matrix are referred to as the singular values. They are typically arranged in descending order, where the largest values are the most relevant.

SVD has been widely adopted for dimensionality reduction in image analysis techniques [8], in addition to being used to extract salient features from either the images’ spatial [28] or wavelet subbands [25]. In this work, the first k singular values were considered in order to extract meaningful information from the chest CT images’ approximation subbands, where k was empirically chosen to be 20.

#### GLCM features

GLCM features, also known as Haralick features, are widely implemented for image texture classification. Initially, the GLCM are created to represent how often specific pixel pairs occur within an image. Next, different order statistical features are computed from these matrices in order to thoroughly describe the image texture. Extracting the GLCM features from the approximation subbands was previously shown to result in reliable performance with the added advantage of reduced time complexity [2]. In this work, twenty-two GLCM features [15, 17, 34] were calculated from the approximation subbands of the chest CT images. Each feature was initially computed from the 0°, 45°, 90°, and 135° directional matrices then averaged in these four directions in order to thoroughly capture textural variations within the image. This approach is similar to that performed in Ref. [34] in which the Haralick features were originally introduced. Table 2 lists the GLCM features considered in this work.

**Table 2:** Summary of the considered GLCM features computed from an image’s approximation subbands

Autocorrelation Cluster Prominence Cluster Shade Contrast Correlation (2) Difference Entropy Difference Variance	Dissimilarity Energy Entropy Homogeneity (2) Information Measures (2) Inverse Difference Normalized Inverse Difference Moment Normalized	Max. Probability Sum Average Sum Entropy Sum Squares Sum Variance

#### Statistical features

Chest CT images including COVID-19 symptoms typically have a cloudier appearance (i.e., less edge related information) than healthy images. Multiresolution analysis separates an image’s edge (high frequency) information within its detail subbands. Three statistical features were thus computed from the detail subbands of the decomposed chest CT images to evaluate their overall cloudiness which are Shannon entropy [16], mean, and standard deviation (*stdev*) as given by the following equations:

2 $${Entropy}_{sb}= - {\sum }_{i=1}^{N}{\left|{C}_{i}\right|}^{2}{\mathit{log}}_{e} {\left|{C}_{i}\right|}^{2}$$

3 $${Mean}_{sb} = \frac{1}{N} {\sum }_{i=1}^{N}{\left|{C}_{i}\right|}$$

4 $${Stdev}_{sb} = \sqrt{\frac{1}{N-1} {\sum }_{i=1}^{N}({Mean}_{sb}- {\left|{C}_{i}\right|)}^{2}}$$

where *N* is the number of coefficients in the considered detail subband (*sb*) and *C*_*i*_ is the detail subband coefficient having index *i*.

In this work, wavelet and contourlet transform-based features are investigated in order to determine which is more suitable for reliable COVID-19 detection from the chest CT images. The introduced features were computed from three decomposition levels of both transforms, then classification performance was compared for the different levels. Specifically, SVD and GLCM features were extracted from the approximation subbands, whereas the statistical features were calculated from the detail subbands. For each image, 20 SVD and 22 GLCM features were extracted from its approximation subband. In addition, three statistical features were calculated from the three and eight detail subbands of the wavelet and contourlet transforms, respectively. The feature vector for each image thus includes a total of 51 features when considering the images’ wavelet decomposition and 66 features when considering their contourlet decomposition.

## Performance metrics

The proposed transform-based COVID-19 detection algorithm was evaluated using six performance metrics that are commonly used in medical AI applications which are [35]:

- **Accuracy**: the percentage of correctly classified cases, both positive and negative, with respect to the entire dataset.
  5 $$Accuracy=\frac{TP+TN}{TP+FP+TN+FN}\times100$$
- **Sensitivity (Recall)**: the percentage of correctly classified positive cases with respect to all the positive cases (i.e., the accuracy of correctly detecting COVID-19 cases).
  6 $$Sensitivity=\frac{TP}{TP+FN}\times100$$
- **Specificity**: the percentage of correctly classified negative cases with respect to all the negative cases (i.e., the accuracy of correctly detecting non-COVID-19 cases).
  7 $$Specificity=\frac{TN}{TN+FP}\times100$$
- **AUC**: In the receiver operating characteristic (ROC) curve, sensitivity and specificity are plotted against each other for different threshold values. A classifier whose performance is no better than random guessing would have an AUC of around 0.5. Better classifiers are those having AUC values that are closer to one.
- **Precision**: the percentage of correctly classified positive cases divided by the total number of positively classified cases.
  8 $$Precision=\frac{TP}{TP+FP}\times100$$
- **F-score**: is the harmonic mean of the model’s precision and sensitivity (recall).
  9 $$F-score=\frac{2(Precision\times Recall)}{Precision+Recall}$$

For Eqs. (5–9), the **TP** (true positive) and **TN** (true negative) values indicate the number of COVID-19 and non-COVID-19 images that are properly classified, respectively. The **FP** (false positive) and **FN** (false negative) are the number of wrongly classified COVID-19 and non-COVID-19, respectively.

## Experiments & results

Two experimental phases were performed in the present study which are: (1) transform analyses and (2) final experiments. In the first phase, several analyses were made to determine the transform and decomposition level most suitable for capturing relevant information related to COVID-19 within the chest CT images. In the second phase, the complete feature vector was first used to compare the performance of several classifiers for the required task. Next, feature selection was implemented to find the best feature set for optimal COVID-19 classification performance. CoDS1 and CoDS2 datasets were utilized for the first and second experimental phases, respectively. The reason why two different datasets were used for each phase was to ensure the generalized and unbiased results of the final experiments. The summary of the performed experiments is given in Table 3.

**Table 3:** Summary of the two experimental phases performed for the development of the proposed transform-based COVID-19 detection algorithm

	Description	Dataset
Phase 1: Transform Analyses	a- Wavelet Filter Analysis: Different wavelet filters were compared in order to determine the basis function that resulted in the best classification performance for the given features	CoDS1
	b- Decomposition Level Analysis: The complete feature vector was computed from three different wavelet and contourlet decomposition levels. Classification performance metrics were then considered to determine the transform and decomposition level most suitable for COVID-19 disease diagnosis	
Phase 2: Final Experiments	a-Classifier Selection: Based on the results of the transform analyses, the complete feature vector was computed from a specific transform level. Results were then compared for five different classifiers	CoDS2
	b-Feature Selection: Irrelevant features were removed from the feature set by applying a feature selection algorithm. The reduced feature vector was then used for the final classification experiments	

MATLAB (MathWorks, Inc., Natick, MA, USA) was used for image preprocessing, multiresolution decomposition, and for the computation of the SVD, GLCM [61], and statistical features. Specifically, wavelet and contourlet decompositions were performed using the Wavelet Toolbox (*dwt* command) [23] and the Contourlet Toolbox (*pdfbdec* command) [18], respectively. Classification and feature selection were performed within the WEKA platform [32]. All computations and processing were made using an Intel Core i7-5500U CPU @2.4 GHz with 16 GB of RAM.

### Transform analyses

#### Wavelet filter analysis

Several wavelet filters (basis functions) exist in literature, each being more suited for a different task [10]. In this work, several wavelet basis functions were compared which are: Haar, Daubechies, and Symlets. Haar is the first and simplest wavelet basis that is represented by a step function. Haar wavelet does not have overlapping windows, by that only reflecting changes between adjacent pixels. Daubechies wavelets are a family of non-symmetric orthogonal functions that are among the most commonly used bases for image texture analysis [56]. They employ overlapping windows, hence are better at capturing edge information within images than the simple Haar wavelet. Symlets are the least-asymmetric wavelets because they are nearly but not exactly symmetrical. Symlets have smoother functions and higher capabilities than the Daubechies wavelets making them more attractive in several image processing applications. For the Daubechies (*dbN*) and Symlets (*symN*) wavelet families, N denotes the number of vanishing moments (where 2N is the number of filter taps) [46]. The Haar wavelet is also referred to as the Daubechies1 (*db1*) wavelet. More information on the different wavelet families can be found in Refs. [56, 58].

Table 4 compares the classification performance of fifteen different wavelet basis functions considering the CoDS1 dataset. For all experiments, the wavelet features were computed from the first decomposition level’s subbands. The k-nearest neighbor (kNN) with tenfold cross validation was used to classify the images. The kNN classifier was chosen as it is easy to tune and gives consistent results. Overall, slight variation in performance was observed among the different wavelet functions. Best performance (i.e., AUC and accuracy) was attained by the *db4* and the *sym10* wavelet basis functions. Since *sym10* achieved a higher sensitivity, i.e., more positive COVID-19 cases were correctly identified, the *sym10* wavelet basis function will be utilized for wavelet decomposition in the next experiments.

**Table 4:** Performance comparison between different wavelet basis functions using the kNN classifier (CoDS1)

	AUC	Accuracy	Sensitivity	Specificity	Precision	F-score
Haar	0.990	95.3	97.1	93.5	93.8	95.4
Db2	0.992	95.3	97.4	93.2	93.6	95.4
Db4	**0.992**	**95.9**	**97.6**	**94.1**	**94.4**	**96.0**
Db6	0.992	95.7	97.7	93.7	94.1	95.8
Db8	0.990	95.7	97.7	93.7	94.0	95.8
Db10	0.990	95.3	97.0	93.7	94.0	95.4
Db15	0.990	95.5	97.0	93.9	94.2	95.6
Db20	0.987	95.2	96.6	93.7	94.0	95.3
Sym2	0.992	95.3	97.4	93.2	93.6	95.4
Sym4	0.991	95.7	97.5	93.8	94.1	95.8
Sym6	0.991	95.5	97.4	93.7	94.0	95.6
Sym8	0.991	95.8	97.9	93.6	93.9	95.9
Sym10	**0.992**	**95.8**	**98.1**	**93.5**	**93.9**	**95.9**
Sym15	0.991	95.7	97.6	93.7	94.1	95.8
Sym20	0.991	96.1	97.8	94.3	94.6	97.8

Bolded values indicate the highest results in the above comparison table

#### Decomposition level analysis

In this analysis, wavelet and contourlet transforms were compared to determine which would be more suitable for COVID-19 detection from chest CT images. For each transform, three-level decomposition was performed, then the complete feature vector was computed from each decomposition level. KNN with tenfold cross validation was used to classify the images as positive or negative COVID-19.

Table 5 summarizes the performance metrics for the six feature vectors computed from the different wavelet and contourlet decomposition levels. Experimental results indicated that considering a specific decomposition level, better performance was attained by the features computed from the contourlet transform. And then for the contourlet transform, better performance was achieved for features calculated from level 1 (L1) followed by those from level 2 (L2). Level 3 (L3) contourlet features, however, gave the lowest results with accuracies being approximately 13% and 10% less than those achieved by the L1 and L2 features, respectively. Level analysis thus showed that the L1-Contourlet feature vector resulted in the best classification performance (AUC = 0.992 and accuracy = 96.3%).

**Table 5:** COVID-19 classification results considering different wavelet and contourlet levels (CoDS1)

	AUC	Accuracy	Sensitivity	Specificity	Precision	F-score
L1-Wavelet	0.992	95.8	98.1	93.5	93.9	95.9
L1-Contourlet	**0.992**	**96.3**	**98.1**	**94.5**	**94.8**	**96.4**
L2-Wavelet	0.974	92.5	93.8	91.1	91.5	92.6
L2-Contourlet	0.982	93.2	95.1	91.3	91.8	93.4
L3-Wavelet	0.877	80.0	84.9	75.0	77.6	81.1
L3-Contourlet	0.907	83.0	87.1	78.8	80.7	83.8

Bolded values indicate the highest results in the above comparison table

In order to preliminary assess the robustness of the proposed L1-Contourlet features, the classification performance was compared to several state-of-the-art deep learning methods from literature (Table 6). Soares et al. [57] and Gaur et al. [31] COVID-19 detection approaches both relied on pre-trained deep networks (TL). Accuracies attained by these methods for the CoDS1 dataset were found to be in the range between 85 and 95%. The L1-Contourlet features implemented in this study gave an accuracy of 96% by that outperforming TL methods by up to 11%. TL approaches rely on standard networks pre-trained on general image classes. These networks are not specifically designed to capture anatomical structures related to the considered disease within the processed medical images. On the other hand, the handcrafted features implemented in this work were carefully chosen to capture the appearance changes within the chest CT images. Specifically, image decomposition into low and high frequency subbands allows for the efficient representation of these changes as a result of the disease progression.

**Table 6:** Performance of several deep learning COVID-19 detection from literature (CoDS1)

Paper	Year	Method	AUC	Accuracy	Sensitivity	Precision	F-score
Soares et al. [57]	2020	AlexNet	0.937	93.75	92.28	94.98	93.61
		VGG16	0.950	94.96	95.43	94.02	94.97
		GoogleNet	0.918	91.73	93.50	90.20	91.82
		ResNet	0.950	94.96	97.15	93.00	95.03
Pathak et al. [49]	2021	MADE-DBM	0.983	98.37	98.87	98.74	98.14
Gaur et al. [31]	2022	WT & DenseNet 12	0.9660	85.05	–	–	85.28
Proposed	2022	L1-Contourlet	**0.992**	**96.30**	**98.10**	**94.80**	**96.40**

* Made-DBM: Memetic Adaptive Differential Evolution—Mixture density network. Bolded values indicate the highest results in the above comparison table

Pathak et al. [49] did not rely on pre-trained networks in their work, instead they used a deep bidirectional long short-term memory network with mixture density network (DBM) whose hyperparameters were tuned using an adaptive evolution algorithm. Although they attain higher accuracies than the L1-Contourlet features implemented in this work, the L1-Contourlet approach achieves a higher AUC, which indicates the robustness of the devised method. Transform-based handcrafted features thus show great potential for the development of reliable automatic COVID-19 detection algorithms.

The aim of the analysis performed in this section was to compare between wavelet and contourlet transforms, as well as to determine the most suitable decomposition level for feature extraction. From this analysis, L1-Contourlet features were found to give the best performance, hence they will be considered for all the upcoming experiments. In the next section, different classifiers will be compared to determine the one best suited for COVID-19 detection. Next, feature selection will be employed to find the best set of features for optimal performance, as well as to investigate the relevance of the different feature types implemented in this work. For these two experiments, L1-Contourelet features will be extracted from a different dataset (i.e., CoDS2) in order to asses the generalizability of the introduced transform-based features.

### Final experiments

#### Classifier selection

Table 7 compares different classifiers for the detection of COVID-19 considering L1-Contourlet features computed from the CoDS2 dataset. The results are presented for five different classifiers which are: kNN, random forest (RF), neural network (NN), support vector machine (SVM) with a linear kernel, and SVM with a radial basis function (rbf) kernel. The parameters of each classifier were tuned for best performance using a grid search algorithm. In all experiments, tenfold cross validation was used. Best performance is shown to be achieved by the kNN and SVM (rbf) classifiers resulting in an AUC of 1.0 and an accuracy of 99.97%. The SVM classifier with an rbf kernel was chosen as it is less computationally demanding the kNN.

**Table 7:** COVID-19 classification results for different classifiers considering the L1-Contourlet features (CoDS2)

Classifier	AUC	Accuracy	Sensitivity	Specificity	Precision	F-score
kNN	1.00	99.97	100.0	99.95	99.95	99.98
RF	1.00	99.81	99.76	99.85	99.84	99.80
NN	0.99	99.91	99.81	100.0	100.0	99.90
SVM (linear)	0.99	98.86	98.78	98.93	98.89	98.84
SVM (rbf)	**1.00**	**99.97**	**99.97**	**99.97**	**99.97**	**99.97**

Bolded values indicate the highest results in the above comparison table

Timing analysis was performed considering the compete feature vector in order to check whether the presented L1-Contourlet method would be suitable for integration in real-time systems. Table 8 summarizes these results considering the CoDS2 dataset which consists of 7,687 images resized to 256 × 256 pixels. The timing analysis showed that in order to compute the complete feature vector, an average of 25 ms were required per image. The tenfold cross validation experiments using the SVM (rbf) classifier required less than 8 s indicating that a single training session was completed in less than a second. The proposed algorithm thus has the advantages of providing reliable performance while remaining computationally inexpensive.

**Table 8:** Timing analysis of the introduced COVID-19 detection algorithm (CoDS2)

Experiment	Details	Time
Feature Extraction	L1-Contourlet complete feature vector (66 features)	25 ms/image
Classification	tenfold cross validation for rbf-SVM classifier	< 8secs

#### Feature selection

Particle swarm optimization (PSO) is an evolutionary search method that was first developed by Kennedy and Eberhart in 1995 [39]. Later, it was adapted to be used for feature selection (FS) and has since been widely used in several machine learning applications. PSO starts with a swarm of random solutions (particles) moving in the search space to find the optimal solution. Each particle’s position is updated based on its own experience in addition to the experience of its neighboring particles. PSO has the advantages of being powerful, having few parameters, as well as being less computationally expensive than other evolutionary search algorithms [62].

L1-Contourlet features include a total of sixty-six textural and statistical features computed from the approximation (42 features) and detail (24 features) subbands. The PSO algorithm was applied to find the most relevant feature set for optimal performance. Twenty-four different features were selected by the PSO algorithm which are summarized in Table 9. The reduced feature set is shown to consist of all feature types including the SVD and GLCM features computed from the approximation subbands, as well as the statistical features calculated from the detail subbands. This indicates relevance of the different transform-based feature types considered in the present study.

**Table 9:** Reduced feature set for COVID-19 detection based on the particle swarm optimization (PSO) technique

Approximation Subband Features		Detail Subbands Features
SVD	GLCM	Statistical
SVD2 SVD5 SVD9 SVD13	Cluster Prominence Energy Homogeneity Max. Probability Sum Average Sum Entropy	Entropy (4) Mean (5) Standard Deviation (5)

Table 10 summarizes the classification results for different combinations of the reduced feature vector. In all experiments, classifications were performed using an SVM (rbf) classifier with tenfold cross validation. Results show that, used separately, statistical features gave the highest results (accuracy = 98.47%) closely followed by the GLCM features (accuracy = 92.14%). The SVD features used alone, however, resulted in the poorest performance (accuracy = 78.31%). Combining two of the feature groups together (i.e., GLCM + SVD, GLCM + statistical, SVD + statistical) led to better performance than when one feature group was solely considered. These results indicate the importance of employing different feature types for reliable COVID-19 diagnosis. Finally, considering all features within the reduced feature vector is shown to give the best performance where an AUC of 1.0 along with accuracy, sensitivity, specificity, precision, and F-score all having a value 100% were achieved.

**Table 10:** Classification results for different combinations of the reduced feature set using the SVM (rbf) classifier (CoDS2)

Features	AUC	Accuracy	Sensitivity	Specificity	Precision	F-score
SVD	0.867	78.31	81.65	75.1	76.0	78.7
GLCM	0.971	92.14	92.9	91.5	91.3	92.1
Statistical	0.998	98.47	98.3	98.6	98.6	98.4
SVD + GLCM	0.998	98.83	98.9	98.7	98.7	98.8
SVD + Statistical	1.00	99.70	99.7	99.7	99.7	99.7
GLCM + Statistical	100	99.99	100	99.97	99.97	99.99
All	**1.00**	**100**	**100**	**100**	**100**	**100**

Bolded values indicate the highest results in the above comparison table

Table 11 summarizes the results of different deep learning approaches from literature that considered the iCTCF dataset. Liu et al. [44] used anomaly weighted images to train several DenseNet networks. A baseline VGG19 network resulted in an accuracy of 95.9%, whereas the different DenseNet networks gave accuracies that were in the range between 96.38% and 97.88%. Liang et al. [42] integrated ResNet and Vision Transformer (ViT) to extract local and global features, respectively. In their work, a weakly supervised module assisted the ViT to better converge. They reported an accuracy of 98.19%. The introduced L1-Contourlet based features resulted in an accuracy of 100% indicating the superiority of the proposed method.

**Table 11:** Performance of several deep learning COVID-19 detection algorithms from literature

Paper	Year	Method	AUC	Accuracy	Sensitivity	Precision	F-score
Liu et al. [44]	2020	VGG19	0.9809	95.90	97.87	96.28	97.07
		DenseNet121	0.9957	97.35	98.80	97.52	98.16
		DenseNet169	0.9996	96.38	98.37	98.06	98.21
		DenseNet201	0.9991	97.88	99.14	97.99	98.56
Liang et al. [42]	2021	FCF-Res	–	98.19	–	–	–
Proposed	2022	**L1-Contourlet**	**1.00**	**100**	**100**	**100**	**100**

Bolded values indicate the highest results in the above comparison table

## Discussion

Transform-based features were scarcely used in literature for the development of COVID-19 detection algorithms from chest CT images. Among the limited traditional works, Yao et al. [63] implemented a wavelet-based COVID-19 detection algorithm in which they only used wavelet-entropy as a feature. They reported an accuracy of 73.95% and an F-score of 73.66%. As for TL based approaches, Matsuyama et al. [47] used the wavelet subbands of the chest CT images as inputs for fine-tuning the ResNet50 network resulting in an accuracy of 92.2% and an F-score of 91.5%. Similarly, Guar et al. [31] input the wavelet subbands to several pre-trained DenseNet networks achieving best accuracy and F-score of 85.5% and 85.25%, respectively. In these works, performance was somewhat limited (i.e., accuracies barely exceeded 92%) indicating that the potential of the transform-based features for COVID-19 detection was not yet fully reached. An interesting work by Dina et al. [53] combined wavelet coefficients alongside textural and deep features derived from several pre-trained networks for chest CT classification. They were able to achieve accuracies of 98.6%, 98%, and 99% for the deep, handcrafted, and combined features, respectively. By that, they showed that deep features (attained from pre-trained networks) and handcrafted features (including the wavelet coefficients) achieved very close performance. Their work shed the light on the relevance of spatial and transform-based handcrafted features, and how they can result in comparable performance to deep learning algorithms.

In this work, a comprehensive study was performed in order to investigate the usefulness of transform-based features for COVID-19 detection from chest CT images. Several analyses were made to determine the transform and decomposition level better suited for COVID-19 detection. L1-Contourlet statistical and textural features were shown to result in accuracies of 96.3% and 100% for two different chest CT datasets. To the best of the author’s knowledge, this work is the first to implement contourlet-based handcrafted features for the development of an automatic COVID-19 diagnosis from chest CT images. The proposed transform-based algorithm was found to outperform several deep learning approaches from literature, specifically those employing TL techniques. Similar findings were reported in Refs. [53] and [12] comparing handcrafted and deep features for COVID-19 detection using CT and X-ray images, respectively. In addition, Ismael et al. [37] showed that simple Shearlet-based features (energy and entropy) computed from chest X-ray images gave better COVID-19 classification performance than ResNet50 regardless of whether or not TL was employed. All these works thus point to the importance of well-designed features for reliable performance.

Multiresolution analysis allows for the separation of an image’s high and low frequency information in its approximation and detail subbands, respectively. Features computed from the different subbands can thus thoroughly capture the changes in the chest CT images due to COVID-19 progression. In addition, contourlet transform provides a high degree of directionality which allows for robust representation of the image characteristics. Accordingly, the superiority of the proposed handcrafted L1-Contourlet features can be attributed to the relevance of the textural and statistical features computed from the different contourlet subbands. Deep pretrained networks commonly employed in medical applications are designed without consideration of the characteristics of the medical images or disease symptoms [3, 43]. Features extracted by these generic deep networks might thus inefficiently represent the different classes resulting in less-than-optimal classification performance.

Another advantage of the introduced transform-based COVID-19 detection algorithm is the reduced computational complexity, specifically in comparison to the deep learning methods. L1-Contourelt features presented in this work were shown to require only a few milliseconds for feature extraction, and less than a second for training. On the other hand, deep networks typically require hours or even days for their training [55]. For example, Liu et al. [44] reported training their deep networks for COVID-19 detection from chest CT images in around 80 h using a Nvidia GeForce GTX 1080 GPU machine. The proposed algorithm thus has the advantage of providing reliable results while remaining computationally inexpensive. Consequently, it can be easily and rapidly re-trained whenever more labeled data is made available. In the COVID-19 pandemic era, this is very useful as every hour counts in saving the lives of hundreds or even thousands of patients [11]. Nevertheless, a limitation of the presented method is that it only classifies the chest CT images into two classes: positive or negative COVID-19. In medical diagnosis, it is typically more desirable to determine the stage of the undergoing disease in order to decide on a suitable treatment plan. In addition, it would be desirable to further investigate its performance over a wide variety of chest CT datasets including real-time environmental data.

## Conclusions

A contourlet-based COVID-19 detection algorithm from chest CT images was introduced and shown to outperform several state-of-the-art methods from literature. A comprehensive study was preliminary performed to determine the transform and decomposition level better suited for the required task. Experiments showed that the handcrafted features computed from the first contourlet decomposition level resulted in the most reliable performance. Feature computations required ~ 25 ms, whereas the rbf SVM classifier was trained in less than a second. Transform-based handcrafted features are thus highly efficient in comparison deep networks which typically require hours for their training. Feature selection was then performed to find the most relevant features, which were shown to include the three different feature types considered in this work. In the final experiments, accuracy, sensitivity, specificity, precision, and F-score of 100% were attained. The proposed contourlet-based approach was shown to outperform various state-of-the-art deep learning methods from literature by up to 11% indicating the superiority of the proposed method. Unlike generic pre-trained deep networks that do not consider specific disease symptoms in the analyzed medical images, handcrafted features have the advantage of efficiently capturing disease manifestations in the images which results in reliable performance. Future work involves designing a deep network that is specifically tailored to capture disease-related features within chest CT images, then comparing its performance to the transform-based method introduced in this study. Furthermore, it would be desirable for clinical purposes to determine the stage of COVID-19, i.e., mild, moderate, or progressive.

## Data Availability

The SARS-CoV-2 dataset (denoted as CoDS1) that supports the findings of this study is a public dataset freely available at https://www.kaggle.com/datasets/plameneduardo/sarscov2-ctscan-dataset. The iCTCF dataset (denoted as CoDS2) that supports the findings of this study is a public dataset freely available at http://ictcf.biocuckoo.cn/.

## References

[1] Abdel-Hamid L (2017) Retinal image analysis using image processing techniques. Ain Shams University

[2] Abdel-Hamid L (2019) Glaucoma detection from retinal images using statistical and textural wavelet features. J Digit Imaging:1–8. 10.1007/s10278-019-00189-0 10.1007/s10278-019-00189-0 PMC7064658 30756264

[3] Abdel-Hamid L (2022). TWEEC: computer-aided glaucoma diagnosis from retinal images using deep learning techniques. Int J Imaging Syst Technol.

[4] Abdel-Hamid L, El-Rafei A, El-Ramly S (2016). Retinal image quality assessment based on image clarity and content. J Biomed Opt.

[5] Abdel-Hamid L, El-Rafei A, El-Ramly S, Michelson G (2018). Performance dependency of retinal image quality assessment algorithms on image resolution: analyses and solutions. Signal Image Video Process.

[6] Aggarwal P, Mishra NK, Fatimah B (2022). COVID-19 image classification using deep learning: advances, challenges and opportunities. Comput Biol Med.

[7] Ahuja S, Panigrahi BK, Dey N et al (2020) Deep transfer learning-based automated detection of COVID-19 from lung CT scan slices. Appl Intell:1–15. 10.1007/s10489-020-01826-w 10.1007/s10489-020-01826-w PMC7440966 34764547

[8] Al-Saffar ZA, Yildirim T (2020). A novel approach to improving brain image classification using mutual information-accelerated singular value decomposition. IEEE Access.

[9] Ali TF, Tawab MA, ElHariri MA (2020). CT chest of COVID-19 patients: what should a radiologist know?. Egypt J Radiol Nucl Med.

[10] Avci E (2008). Comparison of wavelet families for texture classification by using wavelet packet entropy adaptive network based fuzzy inference system. Appl Soft Comput.

[11] Bhuyan HK, Pani SK (2021) Impact of world pandemic “COVID-19” and an assessment of world health management and economics. The internet of medical things: enabling technologies and emerging applications 55

[12] Bozkurt F (2022). A deep and handcrafted features-based framework for diagnosis of COVID-19 from chest x-ray images. Concurr Comput.

[13] Centers for Disease Control and Prevention (CDC) Symptoms of Coronavirus. https://www.cdc.gov/coronavirus/2019-ncov/symptoms-testing/symptoms.html. Accessed 12 Feb 2021

[14] Chen Y, Joshi A (2021). Covid-19 classification based on gray-level co-occurrence matrix and support vector machine BT - COVID-19: prediction, decision-making, and its impacts. Santosh KC.

[15] Clausi DA (2002). An analysis of co-occurrence texture statistics as a function of grey level quantization. Can J Remote Sens.

[16] Coifman RR, Wickerhauser MV (1992). Entropy-based algorithms for best basis selection. IEEE Trans Inf Theory.

[17] Conners RW, Trivedi MM, Harlow CA (1984). Segmentation of a high-resolution urban scene using texture operators. Comput Vis Graph Image Process.

[18] Contourlet Toolbox, MATLAB Central File Exchange. In: Minh Do. https://www.mathworks.com/matlabcentral/fileexchange/8837-contourlet-toolbox. Accessed 8 Feb 2022

[19] Coronavirus Statistics. https://www.worldometers.info/coronavirus/. Accessed 6 Sept 2022

[20] COVID-19 Diagnostic Testing. https://www.mayoclinic.org/tests-procedures/covid-19-diagnostic-test/about/pac-20488900. Accessed 6 Sept 2022

[21] Cucinotta D, Vanelli M (2020). WHO declares COVID-19 a pandemic. Acta Biomed.

[22] Deng J, Dong W, Socher R et al (2009) ImageNet: a large-scale hierarchical image database. In: 2009 IEEE conference on computer vision and pattern recognition, pp 248–255

[23] Discrete 2-D Wavelet Transform. https://www.mathworks.com/help/wavelet/ref/dwt2.html. Accessed 8 Feb 2022

[24] Do MN, Vetterli M (2005). The contourlet transform: an efficient directional multiresolution image representation. IEEE Trans Image Process.

[25] Doğantekin A, Özyurt F, Avcı E, Koc M (2019). A novel approach for liver image classification: PH-C-ELM. Measurement.

[26] Dong D, Tang Z, Wang S (2021). The role of imaging in the detection and management of COVID-19: a review. IEEE Rev Biomed Eng.

[27] Dumic E, Grgic S, Grgic M (2009) New image quality measure based on wavelets. In: Proc.SPIE, pp 72480G

[28] Elelimy E, Mohamed AA (2018) Towards automatic classification of breast cancer histopathological image. In: 2018 13th International Conference on Computer Engineering and Systems (ICCES), pp 299–306

[29] Gao Z, Zheng YF (2008). Quality constrained compression using DWT-based image quality metric. IEEE Trans Circuits Syst Video Technol.

[30] Garg A, Salehi S, La Rocca M (2022). Efficient and visualizable convolutional neural networks for COVID-19 classification using Chest CT. Expert Syst Appl.

[31] Gaur P, Malaviya V, Gupta A (2022). COVID-19 disease identification from chest CT images using empirical wavelet transformation and transfer learning. Biomed Signal Process Control.

[32] Hall M, Frank E, Holmes G (2009). The WEKA data mining software. ACM SIGKDD Explorations Newsl.

[33] Hanif M, Dwivedi UD, Basu M, Gaughan K (2010) Wavelet based islanding detection of DC-AC inverter interfaced DG systems. In: 45th International Universities Power Engineering Conference UPEC2010, pp 1–5

[34] Haralick RM (1979). Statistical and structural approaches to texture. Proc IEEE.

[35] Hicks SA, Strümke I, Thambawita V (2022). On evaluation metrics for medical applications of artificial intelligence. Sci Rep.

[36] Islam Md R, Nahiduzzaman Md (2022). Complex features extraction with deep learning model for the detection of COVID19 from CT scan images using ensemble based machine learning approach. Expert Syst Appl.

[37] Ismael AM, Şengür A (2020). The investigation of multiresolution approaches for chest X-ray image based COVID-19 detection. Health Inf Sci Syst.

[38] Jaiswal A, Gianchandani N, Singh D, et al (2020) Classification of the COVID-19 infected patients using DenseNet201 based deep transfer learning. J Biomol Struct Dyn:1–8 10.1080/07391102.2020.1788642 32619398

[39] Kennedy J, Eberhart R (1995) Particle swarm optimization. In: Proceedings of ICNN’95-international conference on neural networks. IEEE, pp 1942–1948

[40] Khan MA, Azhar M, Ibrar K (2022). COVID-19 classification from chest X-Ray images: a framework of deep explainable artificial intelligence. Comput Intell Neurosci.

[41] Kutlu H, Avcı E (2019). A novel method for classifying liver and brain tumors using convolutional neural networks, discrete wavelet transform and long short-term memory networks. Sensors.

[42] Liang S, Nie R, Cao J (2022). FCF: feature complement fusion network for detecting COVID-19 through CT scan images. Appl Soft Comput.

[43] Litjens G, Kooi T, Bejnordi BE (2017). A survey on deep learning in medical image analysis. Med Image Anal.

[44] Liu Q, Leung CK, Hu P (2020). A two-dimensional sparse matrix profile DenseNet for COVID-19 diagnosis using chest CT images. IEEE Access.

[45] Mary Shyni H, Chitra E (2022). A comparative study of X-RAY and CT images in COVID-19 detection using image processing and deep learning techniques. Comput Methods Programs Biomed Update.

[46] Mathworks (2021) Wavelet Families. https://www.mathworks.com/help/wavelet/ug/wavelet-families-additional-discussion.html#f8-45577. Accessed 10 March 2021

[47] Matsuyama E (2020). A deep learning interpretable model for novel coronavirus disease (COVID-19) screening with chest CT images. J Biomed Sci Eng.

[48] Ning W, Lei S, Yang J et al (2020) iCTCF: an integrative resource of chest computed tomography images and clinical features of patients with COVID-19 pneumonia

[49] Pathak Y, Shukla PK, Arya KV (2021). Deep bidirectional classification model for COVID-19 disease infected patients. IEEE/ACM Trans Comput Biol Bioinform.

[50] Qiu J-J, Wu Y, Hui B (2018). Texture analysis of computed tomography images in the classification of pancreatic cancer and normal pancreas: a feasibility study. J Med Imaging Health Inform.

[51] Radiology Assistant (2020) COVID-19 Imaging Findings. https://radiologyassistant.nl/chest/covid-19/covid19-imaging-findings. Accessed 12 Feb 2021

[52] Radiopaedia (2021) Cases. https://radiopaedia.org/encyclopaedia/cases/all?lang=us. Accessed 28 Jan 2021

[53] Ragab DA, Attallah O (2020). FUSI-CAD: Coronavirus (COVID-19) diagnosis based on the fusion of CNNs and handcrafted features. PeerJ Comput Sci.

[54] Sahidan SI, Mashor MY, Wahab ASW, Abu Osman NA, Ibrahim F, Wan Abas WAB (2008). Local and global contrast stretching for color contrast enhancement on Ziehl-Neelsen tissue section slide images. 4th Kuala Lumpur international conference on biomedical engineering 2008.

[55] Sarker IH (2021). Deep learning: a comprehensive overview on techniques, taxonomy, applications and research directions. SN Comput Sci.

[56] Semler L, Dettori L, Furst J (2005) Wavelet-based texture classification of tissues in computed tomography. In: 18th IEEE Symposium on Computer-Based Medical Systems (CBMS’05). IEEE, pp 265–270

[57] Soares E, Angelov P, Biaso S et al (2020) SARS-CoV-2 CT-scan dataset: a large dataset of real patients CT scans for SARS-CoV-2 identification. medRxiv 2020.04.24.20078584. 10.1101/2020.04.24.20078584

[58] Stolojescu C, Railean I, Moga S, Isar A (2010) Comparison of wavelet families with application to WiMAX traffic forecasting. In: 2010 12th international conference on optimization of electrical and electronic equipment, pp 932–937

[59] Tan H-B, Xiong F, Jiang Y-L (2020). The study of automatic machine learning base on radiomics of non-focus area in the first chest CT of different clinical types of COVID-19 pneumonia. Sci Rep.

[60] Tayarani NM-H (2021). Applications of artificial intelligence in battling against covid-19: a literature review. Chaos Solitons Fractals.

[61] Uppuluri A (2008) GLCM Texture Features. https://www.mathworks.com/matlabcentral/fileexchange/22187-glcm-texture-features. Accessed 8 Dec 2020

[62] Xue B, Zhang M, Browne WN (2014). Particle swarm optimisation for feature selection in classification: novel initialisation and updating mechanisms. Appl Soft Comput.

[63] Yao X, Han J, Joshi A (2021). COVID-19 detection via wavelet entropy and biogeography-based optimization BT - COVID-19: prediction, decision-making, and its impacts. Santosh KC.

[64] Zhou Y, Pei F, Ji M (2020). Sensitivity evaluation of 2019 novel coronavirus (SARS-CoV-2) RT-PCR detection kits and strategy to reduce false negative. PLoS ONE.

